# Cortical and Subcortical Contributions to Neuroplasticity after Repetitive Transspinal Stimulation in Humans

**DOI:** 10.1155/2019/4750768

**Published:** 2019-02-07

**Authors:** Lynda M. Murray, Md. Anamul Islam, Maria Knikou

**Affiliations:** ^1^Klab4Recovery Research Laboratory, Department of Physical Therapy, College of Staten Island, New York, NY 10314, USA; ^2^PhD Program in Biology and Collaborative Neuroscience Program, Graduate Center of The City University of New York, New York, NY 10016, USA

## Abstract

The objectives of this study were to establish cortical and subcortical contributions to neuroplasticity induced by noninvasive repetitive transspinal stimulation in human subjects free of any neurological disorder. To meet our objectives, before and after 40 minutes of transspinal stimulation we established changes in tibialis anterior (TA) motor-evoked potentials (MEPs) in response to paired transcranial magnetic stimulation (TMS) pulses at interstimulus intervals (ISIs) consistent with I-wave periodicity. In order to establish to what extent similar actions are exerted at the spinal cord and motor axons, changes in soleus H-reflex and transspinal evoked potential (TEP) amplitude following transspinal and group Ia afferent conditioning stimulation, respectively, were established. After 40 min of transspinal stimulation, the TA MEP consecutive peaks of facilitation produced by paired TMS pulses were significantly decreased supporting for depression of I-waves. Additionally, the soleus H-reflex and ankle TEP depression following transspinal and group Ia afferent conditioning stimulation was potentiated at intervals when both responses interacted at the spinal cord and nerve axons. These findings support the notion that repetitive transspinal stimulation decreases corticocortical inputs onto corticospinal neurons and promotes a surround inhibition in the spinal cord and nerve axons. This novel method may be a suitable neuromodulation tool to alter excitability at cortical and subcortical levels in neurological disorders.

## 1. Introduction

During the last two decades, electrophysiological studies have clearly demonstrated the reorganization of cortical and corticospinal neural circuits following repetitive stimulation. For example, high-frequency repetitive transcortical stimulation and repetitive paired transcortical stimulation at interstimulus intervals (ISIs) consistent with indirect- (I-) wave periodicity potentiate corticospinal excitability [1–5]. In contrast, low-frequency transcortical stimulation depresses corticospinal excitability [6–8]. Similarly, low-frequency repetitive transspinal stimulation induces neurophysiological changes at both cortical and subcortical levels. Specifically, 40 min of transspinal stimulation delivered at 0.1 Hz decreases the afferent-mediated tibialis anterior (TA) motor-evoked potential (MEP) facilitation, increases the subthreshold transcranial magnetic stimulation- (TMS-) mediated flexor reflex facilitation, and increases corticospinal excitability [9–11]. However, limited evidence exists on how corticocortical influences on corticospinal neurons and subcortical integrated neural signals alter after repetitive transspinal stimulation.

Previous research has demonstrated that information on corticocortical inputs onto corticospinal neurons can be obtained by recording the effects of cortical stimulation. Intrathecal recordings from the epidural space have shown that cortical stimulation produces temporally sequenced descending waves in the corticospinal tract [12, 13]. Early direct- (D-) waves are the result of the direct stimulation of corticospinal neurons, while the subsequent I-waves result from transsynaptic activation of corticospinal neurons by intracortical circuits involving different groups of cortical interneurons [12, 14]. I-waves have distinct neurophysiological properties with respect to their duration and threshold [15], they are influenced differently by inhibitory and facilitatory neurons in the cerebral motor cortex [13, 16–18], and their basic neural characteristics are similar to those observed in epidural responses during *in vivo* recordings of conscious humans [19].

Noninvasive transspinal stimulation over the thoracolumbar enlargement produces concomitantly transspinal-evoked potentials (TEPs) in bilateral ankle and knee muscles with distinct neurophysiological characteristics regarding their latency, duration, shape, and spinally integrated descending or ascending action potentials [9, 20–23]. Summation between TA TEP and TA MEP occurs at latencies that descending motor volleys have depolarized alpha motoneurons, while subtraction of the TEP from the associated MEP reveals depression of corticospinal excitability [9]. In a similar manner, the soleus TEP summates with the soleus M-wave and H-reflex based on the timing of interaction between these responses, while subtraction of the soleus TEP from the conditioned H-reflex or the H-reflex from the conditioned TEP reveals depression of H-reflex or TEP excitability following transspinal or group Ia afferent conditioning stimulation [22, 24].

Collectively, our objective in this study was to establish cortical and subcortical contributions to neuroplasticity induced by noninvasive repetitive transspinal stimulation. To meet our objective, we assessed changes in I-waves, soleus H-reflex amplitude following transspinal conditioning timulation, and TEP amplitude following excitation of group Ia afferents before and after 40 minutes of repetitive low-frequency transspinal stimulation. This approach enabled us to observe changes in corticocortical inputs onto corticospinal neurons and establish to what extent the effects were similar at cortical and subcortical levels.

## 2. Methods

### 2.1. Participants

Fifteen healthy individuals (9 male, 6 female) between 23 and 60 years (32 ± 10.6; mean ± SD) participated in the study. The experimental procedures were approved by the City University of New York Institutional Review Board committee and were conducted in compliance with the Declaration of Helsinki. Each participant signed an informed consent form before enrollment to the study. Subjects were free from neurological or orthopedic disorders. Eligibility to the study was established based on a TMS questionnaire. Individuals with any history of neurological, muscular, or psychiatric disorders were excluded from the study. Furthermore, individuals with any type of implants in the body or pacemakers were also excluded from the study. Poststudy TMS questionnaires revealed no adverse events related to TMS or transspinal stimulation.

### 2.2. Surface EMG Recordings

Compound muscle action potentials were recorded via single bipolar differential surface electrodes (MA300-28, Motion Lab Systems Inc., Louisiana, USA) that were maintained in place by Tegaderm transparent films (3M Healthcare, Minnesota, USA). Compound muscle action potentials were recorded bilaterally from the soleus, TA, medial gastrocnemius (MG), and peroneus longus (PL) muscles. The EMG signals were filtered using a cutoff frequency of 20-1000 Hz (1401 plus running Spike 2; CED Ltd., UK).

### 2.3. Repetitive Transspinal Stimulation for Neuroplasticity

Subjects laid in a supine position with hips and knees flexed at 30°. Two reusable interconnected self-adhering electrodes of 10.2 × 5.1 cm (anode; Uni-Patch™ EP84169, Minnesota, USA) were placed bilaterally on the iliac crests or on the abdominal area based on each subject's comfort level [9, 24, 25]. The T10 vertebra was identified by palpation of spinal processes and anatomical landmarks. A single self-adhesive electrode (cathode; similar type to the anode) was placed parallel to the spinal processes and covered from T10 to L1-2 vertebral levels. The cathode stimulating electrode over the spinal processes was maintained in place via a Tegaderm transparent film and was held under constant pressure via a custom-made pad throughout the experiment. Both cathode and anode electrodes were connected to a DS7A constant current stimulator (Digitimer Ltd., Hertfordshire, UK). Customized scripts written in Spike 2 triggered the constant current stimulator by single 1 ms duration pulses. We delivered for 40 minutes a total of 480 transspinal single pulses at 0.2 Hz with subjects supine. Transspinal stimulation intensities during the stimulation session ranged from 47.6 to 416 mA across subjects (123.87 ± 25.01) and were equivalent to 1.2 times the TA TEP threshold. At these intensities, the blood pressure remained stable and subjects reported no pain or discomfort.

### 2.4. Neurophysiological Recordings before and after 40 Minutes of Transspinal Stimulation

#### 2.4.1. Cortical Neuroplasticity via MEP Peaks of Facilitation

TMS over the left primary motor cortex was delivered with a 110 mm-diameter double-cone coil via two Magstim 200 stimulators with a BiStim module (The Magstim Company Ltd., UK) positioned such that the current flowed from a posterior to an anterior direction. The procedures were similar to those we have previously utilized in our laboratory [9, 11]. Briefly, with the double-cone coil held 1 cm lateral and posterior from Cz, the stimulation intensity was gradually increased from zero, and MEPs recorded from the right soleus, TA, and MG muscles were observed on a digital oscilloscope (TBS1000, Tektronix, Beaverton, OR, USA). When MEPs in the right TA muscle could not be evoked selectively without concomitant MEPs in the soleus and MG muscles at low stimulation intensities, the magnetic coil was moved, and the procedure was repeated. When the optimal position was determined, stimulation intensities increased by 3 MSO and the responses were observed on the oscilloscope. The right TA MEP resting threshold corresponded to the lowest stimulation intensity that induced reproducible MEPs of at least ~50 *μ*V in at least 8 out of 10 consecutive single TMS pulses. TMS was delivered as a single stimulus (test MEP) and as paired stimuli (conditioned MEP). In the paired TMS pulse configuration, the first stimulus (S1) was delivered at 1.27 ± 0.057 (71 ± 10.07% MSO), and the second stimulus (S2) was delivered at 0.85 ± 0.05 (47.27 ± 7.15% MSO) of resting TA MEP threshold across subjects. Stimuli configuration is consistent with the established paired TMS pulse paradigm to assess TMS-induced MEP peaks of facilitation at rest compatible to those recorded from the epidural space [16, 26]. Paired TMS pulses were delivered at the ISIs of 1.1, 1.3, 1.7, 2.3, 2.7, 2.9, 3.5, 4.1, 4.7, and 5.3 ms randomly within and across subjects, and they were selected based on the established peaks of MEP facilitation in awake humans [15, 27, 28]. At each tested ISI, 12 MEPs were recorded at 0.1 Hz, while 24 MEPs were recorded randomly following single-pulse TMS. The group test TA MEP was not statistically significantly different before and after 40 min of transspinal stimulation (area under curve, before: 5.11 ± 2.88 mVms, after: 6.17 ± 2.02 mVms; *p* = 0.16).

#### 2.4.2. Subcortical Neuroplasticity Based on the Conditioning Effects of Transspinal Stimulation and Excitation of Group Ia Afferents on Soleus H-Reflex and TEPs

The soleus H-reflex amplitude modulation following transspinal stimulation and the TEP amplitude modulation recorded bilaterally from flexor and extensor muscles following excitation of soleus group Ia afferents were determined before and after 40 minutes of transspinal stimulation.

The soleus H-reflex was elicited based on experimental methods that we have extensively used in our laboratory [9, 24, 25, 29]. With subjects seated, a stainless steel plate of 4 cm^2^ in diameter was placed and secured proximal to the right patella. A hand-held monopolar stainless steel head electrode was used as a probe to establish the most optimal stimulation site of the right posterior tibial nerve. The optimal site corresponded to the one that at the lowest stimulus intensity an H-reflex could be evoked without the presence of an M-wave, while at increasing stimulation intensities the soleus M-wave had the same shape with the soleus H-reflex. The hand-held electrode was then replaced by a disposable electrode (1800-003, Suretrace, ConMed Corporation, New York, USA) and was maintained under constant pressure throughout the experiment with a custom-made pad and athletic wrap. Subjects were then transferred to a supine position, and the maximal M-wave was determined by increasing progressively the stimulation intensity. We also determined the stimulation intensity that resulted in a soleus H-reflex amplitude ranging from 20 to 30% of the maximal M-wave, which was evoked on the ascending portion of the H-reflex recruitment curve. Transspinal conditioning stimulation was delivered via a similar electrode configuration to that utilized during the delivery of stimulation for 40 minutes. Transspinal stimulation intensity was increased from below threshold levels in order to establish the intensity that resulted in a soleus TEP amplitude ranging from 20 to 30% of the soleus maximal M-wave, which was evoked on the ascending portion of the soleus TEP recruitment curve [10, 22, 25, 29]. Before and after 40 minutes of transspinal stimulation, the soleus TEP amplitude across subjects was 24.4 ± 3.31% and 23.76 ± 2.71% of the soleus maximal M-wave, respectively. Similarly, before and after 40 minutes of transspinal stimulation, the soleus H-reflex amplitude across subjects was 30.59 ± 2.61% and 32.19 ± 2.73% of the soleus maximal M-wave, respectively. These amplitudes support the notion that transspinal and posterior tibial nerve stimulation excited the same alpha motoneurons. For each subject, 30 soleus H-reflexes and TEPs from all leg muscles were recorded at 0.2 Hz under control conditions before and after 40 minutes of transspinal stimulation. Furthermore, soleus H-reflexes and TEPs from all leg muscles were recorded following transspinal and posterior tibial nerve conditioning stimulation before and after 40 minutes of transspinal stimulation. Fifteen conditioned responses at 0.2 Hz were recorded randomly at intervals that ranged from negative 100 to positive 100 ms.

### 2.5. Data Analysis

Test and conditioned MEPs were measured as the area of the full-wave rectified waveform for identical time windows, normalized to the mean amplitude of the homonymous test MEP, and grouped based on TIME and ISI. Normal distribution was tested by the Shapiro-Wilk test (*p* = 0.06) and homogeneity of variances by the Levene test (*p* = 0.94). A repeated measures ANOVA was performed to determine the effect of TIME (before and after 40 minutes of transspinal stimulation) and ISI on the amplitude of the conditioned MEP along with post hoc Bonferroni *t*-tests for multiple comparisons. Furthermore, the normalized TA MEP data from each subject were fitted to a 3-Gaussian model, in which the background EMG level was taken into consideration to estimate the latency and duration of each MEP peak [28, 30, 31]. For each peak *i*, given interstimulus interval *t*_*i*_, amplitude *A*_*i*_, and overall background amplitude *b*_0_ with a Gaussian sigma *σ*_*i*_, all MEP peaks were modeled as shown in equation (1), whereas *y* is the peak amplitude and *t* is the ISI. Data were fitted to the model for each subject separately with 1000 iterations using the Matlab *fit* function, during which a trust-region-reflecting least-squares fit algorithm was applied. The mean of the 1000 fits was chosen for each subject, and 95% confidence intervals were computed across this sample to define the MEP peaks that were significant. A 4th MEP peak was observed only in the average from all subjects and not for each subject separately, and thus it was not included in the analysis.

(1) $$\begin{array}{c} I1\left(t\right)=\left\{\begin{array}{c} b_{0}+\left(A_{1}+b_{0}\right)\times e^{-{\left(t-t1\right)}^{2}/2\sigma_{1}^{2}},\;t<t1,\\ A_{1}\times e^{-{\left(t‐t1\right)}^{2}/2\sigma_{1}^{2}},\;t\geq t1, \end{array}\right.\\ I2\left(t\right)=A_{1}\times e^{-{\left(t-t2\right)}^{2}/2\sigma_{2}^{2}},\\ I3\left(t\right)=A_{3}\times e^{-{\left(t-t3\right)}^{2}/2\sigma_{3}^{2}},\\ y\left(t\right)=100+I1\left(t\right)+I2\left(t\right)+I3\left(t\right)+b_{o}. \end{array}$$

The right soleus TEP and H-reflex were expressed as a percentage of the mean amplitude of the associated test response for each subject separately. Based on the latency and duration of the soleus H-reflex and soleus TEP, both recorded from the right leg, when transspinal stimulation was delivered after stimulation of the mixed tibial nerve, a summation between the H-reflex and TEP is evident at C-T intervals ranging from negative 4 to negative 19 ms in the surface EMG [24]. Furthermore, in some cases soleus H-reflexes evoked at ~28% of the maximal M-wave coincided with small amplitude soleus M-waves. In these cases, the soleus M-wave and the soleus TEP summate in the surface EMG at C-T intervals ranging from 0 to 25 ms when transspinal stimulation is delivered after tibial nerve stimulation [24]. These neuronal phenomena were taken into consideration in order to establish how the effects of transspinal stimulation on soleus H-reflexes and that of muscle spindle group Ia afferent stimulation on TEPs were changed after 40 minutes of transspinal stimulation. Consequently, at C-T intervals that summation was evident, the TEP control amplitude was subtracted from the conditioned soleus H-reflexes, and the soleus H-reflex was subtracted from the conditioned TEP values. Furthermore, M-waves recorded from the right soleus, TA, MG, and PL muscles under control conditions were subtracted from the associated TEPs when a summation between M-waves and TEPs was evident. For all cases, the resultant compound muscle action potentials were normalized to the mean amplitude of the associated unconditioned soleus H-reflex and/or TEP. This analysis was done only for responses recorded from the right leg. Test and conditioned H-reflexes and TEPs were normalized to the mean amplitude of the homonymous test response and grouped based on TIME of testing and C-T interval. A repeated measures ANOVA was performed to determine the effect of TIME (pre-post transspinal stimulation) and C-T interval on the conditioned TEP or H-reflex amplitude. When significance was found, post hoc Bonferroni *t*-tests for multiple comparisons were applied to the data. For all statistical tests, significance was set at *p* < 0.05. Mean and standard error (SE) is indicated in the results.

## 3. Results

### 3.1. Changes in MEP Peaks of Facilitation after 40 min of Transspinal Stimulation

**Figure 1:**
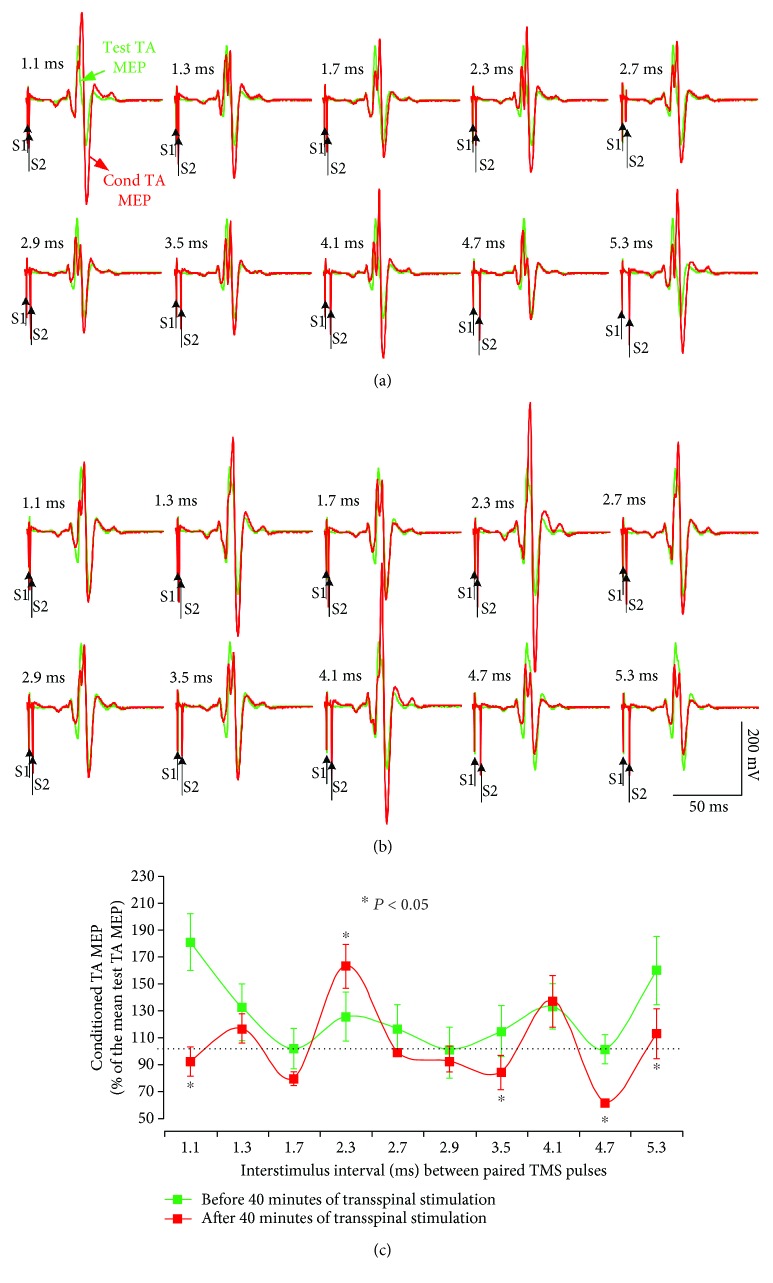
MEP peaks of facilitation. Waveform averages of the right TA MEPs from one representative subject tested while at rest under control conditions (test MEPs; green lines) and following paired TMS pulses (conditioned MEPs; red lines) before (a) and after (b) 40 min of transspinal stimulation. Traces show the average of 24 unconditioned and 12 conditioned MEPs, and arrows indicate the first test stimulus (S1) and second conditioning (S2) stimulus. Group conditioned TA MEPs before and after 40 min of transspinal stimulation for the same subject (c). The abscissa shows the ISI between paired TMS pulses, and the ordinate shows the conditioned MEP size (expressed as a percentage of the test MEP). Error bars indicate SE. ^∗^*p* < 0.05.

Figure 1 demonstrates representative examples of TA MEP waveform averages recorded from the right leg in response to single TMS pulses (unconditioned or test MEP, green lines) and following paired TMS pulses (conditioned MEP, red lines) before (Figure 1(a)) and after (Figure 1(b)) 40 minutes of transspinal stimulation. Note that the first MEP peak before transspinal stimulation occurred at the ISI of 1.1 ms (Figure 1(c)), the second peak occurred at 2.3 ms, and the third peak occurred at 4.1 ms in this subject. After 40 min of transspinal stimulation, the amplitude of the TA MEP upon paired TMS pulses decreased significantly compared to those evoked before transspinal stimulation at the ISIs of 1.1, 3.5, 4.7, and 5.3 ms, while an increase was observed at the ISI of 2.3 ms (Figure 1(c); *p* < 0.05).

**Figure 2:**
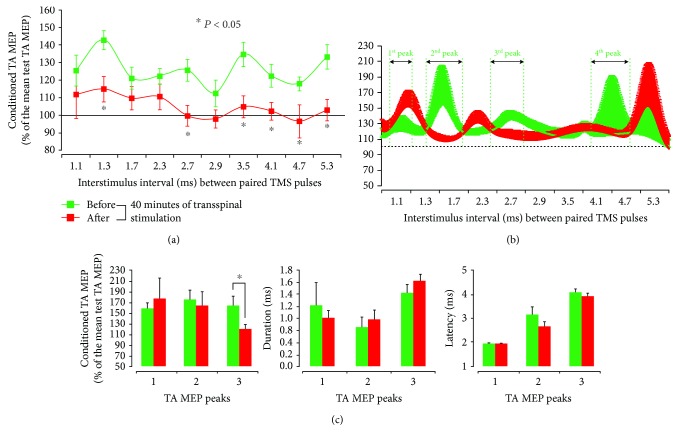
Decrease of MEP peaks of facilitation after 40 minutes of transspinal stimulation. Group data from all subjects showing conditioned MEP (expressed as a percentage of the test MEP) by paired TMS pulses in the resting TA muscle before (green lines) and after (red lines) 40 min of transspinal stimulation for all ISIs tested (a). Curve fitting analysis using a 3-Gaussian model to estimate the number of MEP peaks following paired TMS pulses (b), MEP peak amplitude, onset latency, and duration (c). In (a) and (b), the abscissa shows the ISI between paired TMS pulses, and the ordinate shows the size of the conditioned MEP. In (c), the abscissa shows the TA MEP peaks. Error bars indicate SE. ^∗^*p* < 0.05.

The group of TA MEPs in response to paired TMS pulses before and after 40 min of noninvasive transspinal stimulation over the thoracolumbar enlargement at each ISI is indicated in Figure 2(a). The data fitted to a 3-Gaussian model to estimate the number of MEP peaks are indicated in Figure 2(b). A significant effect of TIME (*F*_1_ = 49.78, *p* < 0.001) and ISI (*F*_9_ = 2.14, *p* = 0.028) but not in their interaction (*F*_9_ = 0.66, *p* = 0.74) for the right TA MEPs suggests that MEPs induced by paired TMS pulses were not of similar amplitude across ISIs, and that they were decreased after repetitive transspinal stimulation (Figure 2(a)). Specifically, the post hoc Bonferroni *t*-test for multiple comparisons showed that TA MEPs upon paired TMS pulses were significantly different before and after transspinal stimulation at the ISIs of 1.3, 2.7, 3.5, 4.1, 4.7, and 5.3 ms (*p* < 0.05 for all), supporting the notion that repetitive transspinal stimulation depresses I1-, I2-, and I3-waves. Based on Gaussian fit to the data, the conditioned MEP peak 3 was decreased (*p* = 0.02; Figure 2(c)), while a tendency for the first MEP peak to increase after transspinal stimulation was observed. No significant changes were observed in the latency or duration of all MEP peaks (Figure 2(c)).

### 3.2. Changes in the Effects of Transspinal Conditioning Stimulation on the Soleus H-Reflex after 40 Minutes of Transspinal Stimulation

**Figure 3:**
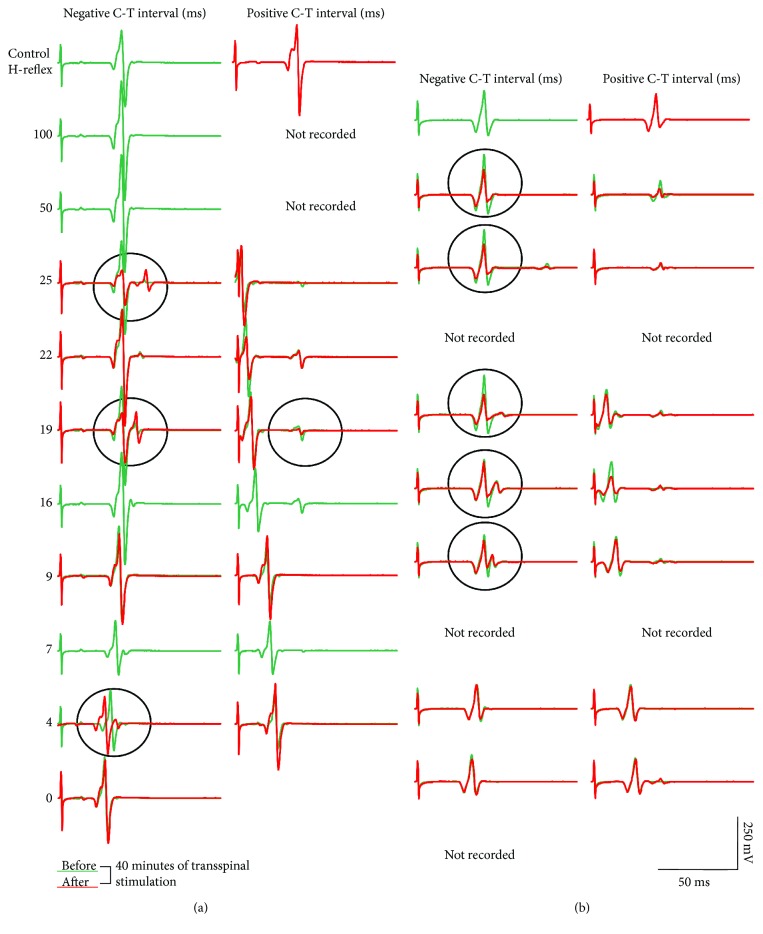
Soleus H-reflex depression by transspinal conditioning stimulation before and after 40 minutes of transspinal stimulation. Waveform averages of the right soleus H-reflex from two representative subjects under control conditions and following transspinal conditioning stimulation before (green lines) and after (red lines) 40 min of transspinal stimulation. All traces are shown as captured, without subtraction, to counteract summation of soleus H-reflex and soleus TEP at C-T intervals when this phenomenon is observed. A negative C-T interval denotes that transspinal stimulation was delivered after stimulation of soleus group Ia afferents. Circled traces indicate potentiation of soleus H-reflex depression by transspinal conditioning stimulation after 40 min of transspinal stimulation. Traces show the average of 15 unconditioned and conditioned soleus H-reflexes.

Figure 3 demonstrates soleus H-reflex waveform averages recorded from the right leg before and after 40 minutes of transspinal stimulation from two representative subjects under control conditions and following transspinal conditioning stimulation. Negative C-T intervals correspond to cases when transspinal conditioning stimulation was delivered after posterior tibial nerve stimulation. For all cases, soleus H-reflex waveform averages are shown as depicted on the surface EMG. It is apparent that the early-latency long-lasting soleus H-reflex depression following transspinal conditioning stimulation was significantly increased after 40 minutes of transspinal stimulation.

**Figure 4:**
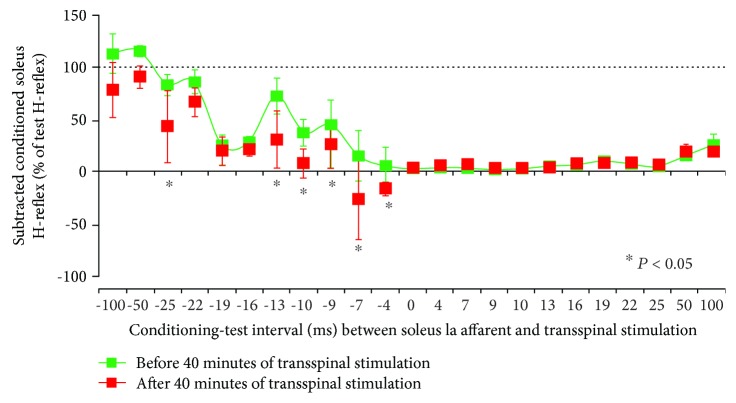
Potentiation of soleus H-reflex depression by transspinal conditioning stimulation after 40 minutes of transspinal stimulation. Group data from all subjects show the amplitude of the conditioned soleus H-reflexes (expressed as a percentage of the test H-reflex) in response to transspinal conditioning stimulation over the thoracolumbar region before and after 40 min of transspinal stimulation. The abscissa shows the C-T interval between transspinal stimulation and excitation of group Ia afferents following posterior tibial nerve stimulation. Error bars denote the SE. ^∗^*p* < 0.05.

Before and after 40 min of transspinal stimulation over the thoracolumbar enlargement, the average amplitude of the soleus H-reflex in response to transspinal conditioning stimulation at each C-T interval from all subjects is indicated in Figure 4. The conditioned soleus H-reflexes are normalized to the mean amplitude of the test H-reflex. A significant effect of TIME (*F*_1_ = 29.38, *p* < 0.001), C-T interval (*F*_22_ = 33.86, *p* < 0.001), and in their interaction (*F*_22_ = 2.75, *p* < 0.001) supports for the potentiation of soleus H-reflex depression when conditioned by transspinal stimulation after 40 minutes of transspinal stimulation (Figure 4). Specifically, the post hoc Bonferroni *t*-test for multiple comparisons showed that the conditioned soleus H-reflex before and after 40 minutes of transspinal stimulation was significantly different at the negative C-T intervals of 25, 13, 10, 9, 7, and 4 ms (*p* < 0.05 for all). The soleus H-reflex depression at positive C-T intervals remained unaltered after 40 minutes of transspinal stimulation (Figure 4).

### 3.3. Changes in the Amplitude of TEPs when Conditioned by Excitation of Group Ia Afferents after 40 Minutes of Transspinal Stimulation

**Figure 5:**
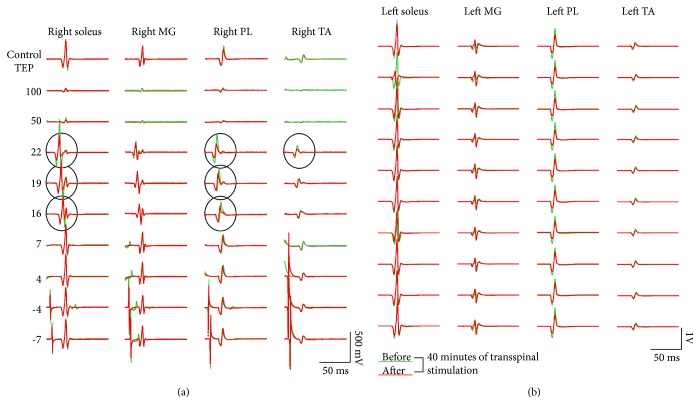
TEP depression by soleus muscle spindle group Ia afferent stimulation before and after 40 minutes of transspinal stimulation. Waveform averages of TEPs from one representative subject under control conditions and following transspinal conditioning stimulation before (green lines) and after (red lines) 40 min of transspinal stimulation. All traces are shown as captured, without subtraction, to counteract summation between soleus H-reflex and soleus TEP at C-T intervals when this phenomenon is observed. Circled traces indicate potentiation of TEP depression by soleus muscle spindle group Ia afferent conditioning stimulation after 40 min of transspinal stimulation.

Representative examples of TEP waveform averages recorded before and after 40 minutes of transspinal stimulation are indicated in Figure 5. TEP waveform averages correspond to TEPs recorded under control conditions and following conditioning excitation of soleus group Ia afferents at negative and positive C-T intervals. In this case, a negative C-T interval corresponds to the interval when transspinal stimuli are delivered before posterior tibial nerve stimulation. TEP waveform averages are shown as depicted on the surface EMG. It is evident that after 40 minutes of transspinal stimulation, the TEP depression induced by excitation of group Ia afferents was increased (see circled traces in Figure 5(a)).

**Figure 6:**
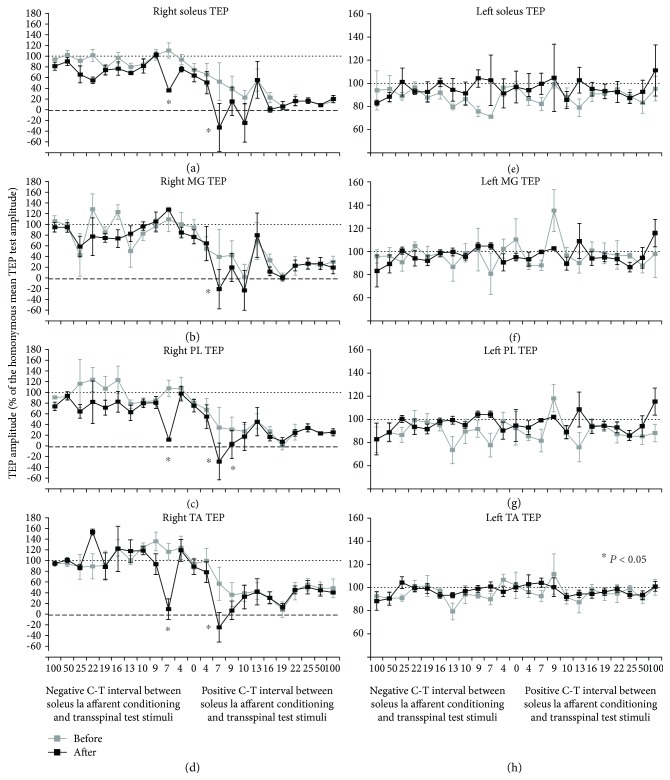
Potentiation of TEP depression by muscle spindle group Ia afferent conditioning stimulation after 40 minutes of transspinal stimulation. Group data from all subjects show the amplitude of TEP recorded from the right (a-d) and left (e-h) ankle muscles following soleus muscle spindle Ia afferent conditioning stimulation before and after 40 min of transspinal stimulation. The abscissa shows the C-T interval between these two stimuli, and the ordinate shows the size of the TEP (expressed as a percentage of the homonymous test TEP). Error bars denote the SE. ^∗^*p* < 0.05.

The normalized TEPs recorded from each muscle and all subjects following excitation of group Ia afferents before and after 40 minutes of transspinal stimulation over the thoracolumbar enlargement is indicated in Figure 6. The C-T interval is denoted on the abscissa, and the conditioned TEPs are presented as a percentage of the homonymous test response value. For the right soleus TEP, a significant effect of TIME (*F*_1_ = 26.28, *p* < 0.001), C-T interval (*F*_22_ = 18.97, *p* < 0.001) and in their interaction (*F*_22_ = 1.79, *p* < 0.001) supports for potentiation of soleus TEP depression following excitation of group Ia afferents after 40 minutes of transspinal stimulation (Figure 6(a)). Specifically, the post hoc Bonferroni *t*-test for multiple comparisons showed that the conditioned right soleus TEP before and after transspinal stimulation was significantly different at the C-T intervals of positive and negative 7 ms (*p* < 0.05 for both). An increase of depression was also found for the right MG TEP, but potentiation of depression occurred only at the positive C-T interval of 7 ms (TIME: *F*_1_ = 3.45, *p* = 0.04; C-T interval: *F*_22_ = 14.66, *p* < 0.001). Similarly, for the right PL TEP an increase of depression was apparent at the C-T intervals of -7, 7, and 9 ms (TIME: *F*_1_ = 18.69, *p* < 0.001; C-T interval: *F*_22_ = 14.65, *p* < 0.001). Lastly, repetitive transspinal stimulation potentiated the TEP depression in the antagonistic ipsilateral TA muscle at the C-T intervals of positive and negative 7 ms (*p* < 0.05 for both). No significant differences were found before or after 40 minutes of transspinal stimulation for TEPs being conditioned by stimulation of group Ia afferents and recorded from the left ankle muscles (Figures 6(e)-6(h)). The results regarding the conditioning effects observed at baseline are consistent with our recent findings that excitation of ipsilateral group Ia afferents does not alter the TEP amplitude of the contralateral ankle muscles [24].

## 4. Discussion

This study delineates the neuroplasticity mechanisms underlying repetitive transspinal stimulation over the thoracolumbar enlargement, the location of spinal neuronal networks for leg motor control. We found that 40 minutes of transspinal stimulation reduced the amplitude of cortical I-waves, increased the soleus H-reflex depression following transspinal conditioning stimulation, and increased the TEP depression following excitation of group Ia afferents. Consequently, there is support for the increase in motor surround inhibition by low-frequency transspinal stimulation.

In this study, a paired-pulse TMS paradigm was used to study changes in TMS-induced MEP facilitation peaks in a flexor ankle muscle after repetitive low-frequency transspinal stimulation over the thoracolumbar region. Results indicated that MEP facilitation peaks following paired TMS pulses (Figures 2(b) and 2(c)) occurred at ISI consistent with those reported in the literature [27, 28]. The MEP facilitation peaks in response to paired TMS pulses are the result of a summation of excitatory postsynaptic potentials elicited by the first suprathreshold TMS pulse and depolarization of interneurons that are at a subliminal fringe elicited by the consecutive second subthreshold TMS pulse [27], and thus they are fully cortically mediated. The TA MEP facilitation peaks were significantly decreased after 40 min of transspinal stimulation, supporting the notion that repetitive transspinal stimulation depresses cortically induced I-waves. A question that arises is related to the neuronal pathways of mediating such action. Based on the decreased afferent-mediated MEP facilitation after 40 min of transspinal stimulation [11], increased intracortical facilitation after repetitive peripheral magnetic stimulation [32], and on the consideration that EEG activity in response to peripheral nerve stimulation occurs at frequencies consistent with cortical spikes [33], we suggest that transspinal stimulation affected directly the activity of cortical interneurons via thalamocortical axons. This is further supported by the altered cortical feedback mechanisms when transspinal stimulation was delivered before and not after transcortical stimulation in a repetitive paired associative paradigm [10]. Potentiation of cortical inhibitory interneurons and/or decreased activity of cortical facilitatory interneurons constitute potential mechanisms for I-wave depression after repetitive transspinal stimulation. Short interval intracortical inhibition facilitates short interval intracortical facilitation of circuits responsible for I-waves [34], while replacement of S2 with anodal or cathodal transcranial electrical stimulation failed to produce MEP facilitation [26]. Furthermore, based on the transient suppression of MEP peak facilitation by *γ*-aminobutyric acid-enhancing drugs in humans [17], and that intracortical inhibition is prominent and linearly related to I-waves [27, 35], we theorize that repetitive transspinal stimulation depressed MEP peak facilitation via intracortical inhibitory circuits decreasing the amplitude of I-waves.

In addition to I-wave depression, 40 minutes of low-frequency transspinal stimulation increased the soleus H-reflex depression as a result of transspinal conditioning stimulation (Figure 4). At baseline, transspinal conditioning stimulation induced a short-latency, long-lasting soleus H-reflex depression (Figure 4), which is consistent with our previous observations [21, 23, 24]. Based on the latency (10-12 ms) and duration (5-8 ms) of the segmental potentials recorded intrathecally following posterior tibial nerve stimulation in humans [36], when posterior tibial nerve stimulation was delivered before transspinal stimulation at intervals ranging from 22 to 7 ms, soleus Ia afferents had already monosynaptically depolarized soleus alpha motoneurons before transspinal stimulation could have produced motoneuron depolarization via transsynaptic activation, and thus reflex inhibition was exerted at the nerve axons. The potentiation of the soleus H-reflex inhibition after repetitive transspinal stimulation is consistent with the shift of excitation thresholds of Ia afferents and motor axons after transspinal-transcortical paired stimulation [10]. Additionally, prolonged decreases in axonal excitability are reported after 10 min of median nerve stimulation at 8 Hz [37]. The decreased axonal excitability was associated with a reduction in refractoriness and a decrease in the strength-duration time constant, both consistent with axonal hyperpolarization [37], a phenomenon commonly observed after muscle contractions [38]. Based on this evidence, we suggest that repetitive transspinal stimulation promoted hyperpolarization of group Ia afferents through changes in anodal and internodal ion channels. Collectively, our findings suggest that repetitive transspinal stimulation induces selective changes in the ability of group Ia afferents to depolarize soleus motoneurons by altering the excitability of Ia afferents or the number of active motoneurons [29, 39].

Similar neuromodulation effects to that of the soleus H-reflex were also observed after 40 minutes of transspinal stimulation in TEPs recorded from ankle flexor and extensor muscles following conditioning excitation of group Ia afferents. Specifically, the depression of TEPs in response to stimulation of muscle spindle group Ia afferents was potentiated at the interval of 7 ms, while TEP depression reemerged after 40 minutes of transspinal stimulation at the C-T interval of -7 ms for most TEPs (Figure 6). These effects were observed in the ipsilateral TEPs and not in the contralateral TEPs, which remained unaltered before or after 40 minutes of transspinal stimulation.

The short-latency, long-lasting TEP depression induced by excitation of group Ia afferents observed at baseline (before 40 minutes of transspinal stimulation) is related to the interaction between TEPs and H-reflexes. Based on collision experiments, it was proposed that transspinal stimulation excites nerve roots at their exit from the spinal canal [40, 41] and large-diameter proprioceptive afferent fibers [42–44], resulting in bilateral contractions of knee and ankle muscles. TEP depression following group Ia afferent excitation started when group Ia afferents were excited 4 or 7 ms after transspinal stimulation, lasting up to 100 ms (Figure 6). At the C-T intervals of 4 or 7 ms, neural interactions between action potentials occur likely at the nerve axons because afferent volleys have not reached the spinal motoneurons. Thus, it is likely that an occlusion occurred between the two propagated potentials at the peripheral mixed nerve fibers to produce the TEP depression. In contrast, Ia afferent volleys at the C-T interval of 10 ms and beyond reach the spinal motoneurons and could potentially alter the TEP amplitude via activation of spinal inhibitory interneuronal circuits.

We theorize that potentiation of TEP depression following excitation of group Ia afferents after 40 minutes of transspinal stimulation is the result of hyperpolarization in nerve axons or nerve terminals, and increased depolarization in the presynaptic endings through an increased GABAergic inhibitory hyperpolarizing neurotransmission [45]. Possible spinal inhibitory interneurons include Renshaw cells, possible spinal interneurons include Renshaw cells, and reciprocal and presynaptic inhibitory interneurons [46]. Our theory is based on the increased amplitude of the soleus H-reflex homosynaptic depression and postactivation depression after transspinal-transcortical paired stimulation [10]. However, direct recordings of excitatory postsynaptic potentials and primary afferent depolarizing currents upon transspinal stimulation are needed to elucidate the exact mechanism(s) of action.

## 5. Limitations of the Study

In this study, transspinal stimulation was delivered in a single session for 40 minutes. Because the neurophysiological tests were not conducted at different times after cessation of stimulation and multiple sessions were not delivered, future studies are needed to assess the time course of the effects incorporating multiple sessions of transspinal stimulation. Furthermore, stimulation was delivered as single 1 ms pulses at 0.2 Hz based on previous stimulation paradigms we have used in our laboratory [10, 11, 25]. On the basis that stimulation-based neuroplasticity is frequency dependent [47], different frequencies warrant further investigation. Lastly, to study changes in I-waves we used ISIs ranging from 1.1 to 5.3 ms that did not increment in a linear fashion, while longer ISIs during which peaks of MEP facilitation have been demonstrated [18] were not tested. This probably resulted in a lack of characterization of the effects on late I4- and I5-waves [18], but the ISIs used for the I1-wave (1.1-2 ms), I2-wave (2-4 ms), and I3-wave (4.0-5.3 ms) in the 3-Gaussian model are consistent with those used elsewhere [15, 28]; however, the model was not affected by the number of ISIs since the model detected changes in MEP peaks of facilitation reflecting I1-, I2-, and I3-waves.

## 6. Functional Significance

We found that low-frequency repetitive transspinal stimulation increases motor surround inhibition, as evidenced by depression of cerebral cortical circuits controlling descending motor I-waves, potentiation of H-reflex depression in response to transspinal conditioning stimulation, and potentiation of TEP depression in response to Ia afferent conditioning. The latter two neural adaptations occurred both at spinal cord and nerve axons. Because I-waves play a pivotal role in determining the firing rates of spinal motoneurons [30], motor surround inhibition is decreased in dystonia [48], and hyperreflexia along with impaired spinal inhibitory actions characterizes upper motor neuron lesions [49]; therefore, this intervention may have beneficial effects in different types of neurological disorders.

## Data Availability

The data used to support the findings of this study are available on request from Dr. Maria Knikou at Maria.Knikou@csi.cuny.edu.

## Abbreviations

I-waves: Indirect waves
MEPs: Motor-evoked potentials
MG: Medial gastrocnemius
PL: Peroneus longus
TA: Tibialis anterior
TEPs: Transspinal-evoked potentials
TMS: Transcranial magnetic stimulation.

## References

[1] Pascual-Leone A., Valls-Solé J., Wassermann E. M., Hallett M. (1994). Responses to rapid-rate transcranial magnetic stimulation of the human motor cortex. *Brain*.

[2] Stefan K., Kunesch E., Cohen L. G., Benecke R., Classen J. (2000). Induction of plasticity in the human motor cortex by paired associative stimulation. *Brain*.

[3] Thickbroom G. W., Byrnes M. L., Edwards D. J., Mastaglia F. L. (2006). Repetitive paired-pulse TMS at I-wave periodicity markedly increases corticospinal excitability: a new technique for modulating synaptic plasticity. *Clinical Neurophysiology*.

[4] Cash R. F., Benwell N. M., Murray K., Mastaglia F. L., Thickbroom G. W. (2009). Neuromodulation by paired-pulse TMS at an I-wave interval facilitates multiple I-waves. *Experimental Brain Research*.

[5] Murray L. M., Nosaka K., Thickbroom G. W. (2011). Interventional repetitive I-wave transcranial magnetic stimulation (TMS): the dimension of stimulation duration. *Brain Stimulation*.

[6] Chen R., Classen J., Gerloff C. (1997). Depression of motor cortex excitability by low-frequency transcranial magnetic stimulation. *Neurology*.

[7] Wolters A., Sandbrink F., Schlottmann A. (2003). A temporally asymmetric Hebbian rule governing plasticity in the human motor cortex. *Journal of Neurophysiology*.

[8] Di Lazzaro V., Profice P., Pilato F., Dileone M., Oliviero A., Ziemann U. (2010). The effects of motor cortex rTMS on corticospinal descending activity. *Clinical Neurophysiology*.

[9] Knikou M. (2014). Transpinal and transcortical stimulation alter corticospinal excitability and increase spinal output. *PLoS One*.

[10] Dixon L., Ibrahim M. M., Santora D., Knikou M. (2016). Paired associative transspinal and transcortical stimulation produces plasticity in human cortical and spinal neuronal circuits. *Journal of Neurophysiology*.

[11] Knikou M., Dixon L., Santora D., Ibrahim M. M. (2015). Transspinal constant-current long-lasting stimulation: a new method to induce cortical and corticospinal plasticity. *Journal of Neurophysiology*.

[12] Patton H. D., Amassian V. E. (1954). Single and multiple-unit analysis of cortical stage of pyramidal tract activation. *Journal of Neurophysiology*.

[13] Di Lazzaro V., Profice P., Ranieri F. (2012). I-wave origin and modulation. *Brain Stimulation*.

[14] Di Lazzaro V., Ziemann U. (2013). The contribution of transcranial magnetic stimulation in the functional evaluation of microcircuits in human motor cortex. *Frontiers in Neural Circuits*.

[15] Cirillo J., Perez M. A. (2015). Subcortical contribution to late TMS-induced I-waves in intact humans. *Frontiers in Integrative Neuroscience*.

[16] Tokimura H., Ridding M. C., Tokimura Y., Amassian V. E., Rothwell J. C. (1996). Short latency facilitation between pairs of threshold magnetic stimuli applied to human motor cortex. *Electroencephalography and Clinical Neurophysiology*.

[17] Ziemann U., Tergau F., Wischer S., Hildebrandt J., Paulus W. (1998). Pharmacological control of facilitatory I-wave interaction in the human motor cortex. A paired transcranial magnetic stimulation study. *Electroencephalography and Clinical Neurophysiology*.

[18] Kallioniemi E., Savolainen P., Järnefelt G., Koskenkorva P., Karhu J., Julkunen P. (2018). Transcranial magnetic stimulation modulation of corticospinal excitability by targeting cortical I-waves with biphasic paired-pulses. *Brain Stimulation*.

[19] Rusu C. V., Murakami M., Ziemann U., Triesch J. (2014). A model of TMS-induced I-waves in motor cortex. *Brain Stimulation*.

[20] Maruyama Y., Shimoji K., Shimizu H., Kuribayashi H., Fujioka H. (1982). Human spinal cord potentials evoked by different sources of stimulation and conduction velocities along the cord. *Journal of Neurophysiology*.

[21] Knikou M. (2013). Neurophysiological characteristics of human leg muscle action potentials evoked by transcutaneous magnetic stimulation of the spine. *Bioelectromagnetics*.

[22] Knikou M. (2013). Neurophysiological characterization of transpinal evoked potentials in human leg muscles. *Bioelectromagnetics*.

[23] Einhorn J., Li A., Hazan R., Knikou M. (2013). Cervicothoracic multisegmental transpinal evoked potentials in humans. *PLoS One*.

[24] Knikou M., Murray L. M. (2018). Neural interactions between transspinal evoked potentials and muscle spindle afferents in humans. *Journal of Electromyography and Kinesiology*.

[25] Knikou M. (2017). Spinal excitability changes after transspinal and transcortical paired associative stimulation in humans. *Neural Plasticity*.

[26] Ziemann U., Tergau F., Wassermann E. M., Wischer S., Hildebrandt J., Paulus W. (1998). Demonstration of facilitatory I wave interaction in the human motor cortex by paired transcranial magnetic stimulation. *The Journal of Physiology*.

[27] Hanajima R., Ugawa Y., Terao Y. (2002). Mechanisms of intracortical I-wave facilitation elicited with paired-pulse magnetic stimulation in humans. *The Journal of Physiology*.

[28] Cirillo J., Calabro F. J., Perez M. A. (2016). Impaired organization of paired-pulse TMS-induced I-waves after human spinal cord injury. *Cerebral Cortex*.

[29] Knikou M. (2008). The H-reflex as a probe: pathways and pitfalls. *Journal of Neuroscience Methods*.

[30] Thickbroom G. W. (2011). A model of the contribution of late I-waves to *α*-motoneuronal activation: implications for paired-pulse TMS. *Brain Stimulation*.

[31] Delvendahl I., Lindemann H., Jung N. H., Pechmann A., Siebner H. R., Mall V. (2014). Influence of waveform and current direction on short-interval intracortical facilitation: a paired-pulse TMS study. *Brain Stimulation*.

[32] Baker S. N., Gabriel C., Lemon R. N. (2003). EEG oscillations at 600 Hz are macroscopic markers for cortical spike bursts. *The Journal of Physiology*.

[33] Wagle-Shukla A., Ni Z., Gunraj C. A., Bahl N., Chen R. (2009). Effects of short interval intracortical inhibition and intracortical facilitation on short interval intracortical facilitation in human primary motor cortex. *The Journal of Physiology*.

[34] Nakamura H., Kitagawa H., Kawaguchi Y., Tsuji H. (1997). Intracortical facilitation and inhibition after transcranial magnetic stimulation in conscious humans. *The Journal of Physiology*.

[35] Ertekin C. (1976). Studies on the human evoked electrospinogram. I. The origin of the segmental evoked potentials. *Acta Neurologica Scandinavica*.

[36] Kiernan M. C., Lin C. S. Y., Burke D. (2004). Differences in activity-dependent hyperpolarization in human sensory and motor axons. *The Journal of Physiology*.

[37] Vagg R., Mogyoros I., Kiernan M. C., Burke D. (1998). Activity-dependent hyperpolarization of human motor axons produced by natural activity. *The Journal of Physiology*.

[38] Burke D., Kiernan M. C., Bostock H. (2001). Excitability of human axons. *Clinical Neurophysiology*.

[39] Mills K. R., Murray N. M. F. (1986). Electrical stimulation over the human vertebral column: which neural elements are excited?. *Electroencephalography and Clinical Neurophysiology*.

[40] Ugawa Y., Rothwell J. C., Day B. L., Thompson P. D., Marsden C. D. (1989). Magnetic stimulation over the spinal enlargements. *Journal of Neurology, Neurosurgery, and Psychiatry*.

[41] Danner S. M., Hofstoetter U. S., Ladenbauer J., Rattay F., Minassian K. (2011). Can the human lumbar posterior columns be stimulated by transcutaneous spinal cord stimulation? A modeling study. *Artificial Organs*.

[42] Hunter J. P., Ashby P. (1994). Segmental effects of epidural spinal cord stimulation in humans. *The Journal of Physiology*.

[43] Maertens de Noordhout A., Rothwell J. C., Thompson P. D., Day B. L., Marsden C. D. (1988). Percutaneous electrical stimulation of lumbosacral roots in man. *Journal of Neurology, Neurosurgery, and Psychiatry*.

[44] Eccles J. C., Kostyuk P. G., Schmidt R. F. (1962). The effect of electric polarization of the spinal cord on central afferent fibres and on their excitatory synaptic action. *The Journal of Physiology*.

[45] Côté M. P., Murray L. M., Knikou M. (2018). Spinal control of locomotion: individual neurons, their circuits and functions. *Frontiers in Physiology*.

[46] Doussau F., Schmidt H., Dorgans K., Valera A. M., Poulain B., Isope P. (2017). Frequency-dependent mobilization of heterogeneous pools of synaptic vesicles shapes presynaptic plasticity. *eLife*.

[47] Kassavetis P., Sadnicka A., Saifee T. A. (2018). Reappraising the role of motor surround inhibition in dystonia. *Journal of the Neurological Sciences*.

[48] Nielsen J. B., Crone C., Hultborn H. (2007). The spinal pathophysiology of spasticity—from a basic science point of view. *Acta Physiologica*.

[49] Gallasch E., Christova M., Kunz A., Rafolt D., Golaszewski S. (2015). Modulation of sensorimotor cortex by repetitive peripheral magnetic stimulation. *Frontiers in Human Neuroscience*.

